# Novel Pyrazole‐4‐Carbaldehyde Derivatives Having Potent COX‐2 Inhibition and Anti‐Tubercular Activity: Microwave‐Assisted Green Synthesis and Molecular Docking

**DOI:** 10.1002/jbt.71113

**Published:** 2026-09-17

**Authors:** Megha Kokane, Dnyandev Bhosale, Dattatraya Raut, Pushpa Hadimani, Ashwini Narale, Prafulla Choudhari, Anjana Lawand

**Affiliations:** ^1^ School of Chemical Sciences Punyashlok Ahilyadevi Holkar Solapur University Solapur Maharashtra India; ^2^ Department of Chemistry Pratapsinh Mohite‐Patil Mahavidyalaya Karmala Solapur India; ^3^ Vishwasrao Naik Arts Commerce, and Baba Naik Science Mahavidyalaya, Shirala District‐Sangli Maharashtra state India; ^4^ Department of Pharmaceutical Chemistry Bharati Vidyapeeth College of Pharmacy Kolhapur Maharashtra state India

**Keywords:** anti‐inflammatory, anti‐tubercular, microwave‐assisted green synthesis, molecular docking, pyrazole‐4‐carbaldehydes

## Abstract

A series of 1‐(4‐Chorophenyl)‐3Aryl‐1‐*H*‐pyrazole‐4‐carbaldehyde derivatives were synthesized under the microwave irradiation (MW) technique. IR, ^1^H‐NMR, ^13^C‐NMR, and mass spectroscopic techniques were used to confirm the structure of the synthesized compounds. The newly synthesized Pyrazole aldehydes were evaluated for their in vitro anti‐inflammatory and anti‐tubercular activity. Among them, the compounds **4c** and **4e** exhibited significant reduction of LPS‐induced COX‐2 protein levels across all tested concentrations. Whereas compounds **4f** and **4j** exhibited sensitivity to tuberculosis at the lowest concentration of 6.25 µg/mL, which was comparable to that of the standard drug pyrazinamide. Molecular docking against the Crystal structure of diclofenac bound to the cyclooxygenase active site of COX‐2 (PDB ID: 5KIR) also supports the experimental results. The molecular docking studies have demonstrated their better‐fit potential as anti‐inflammatory agents.

## Summary

- A series of 1‐(4‐chlorophenyl)‐3‐aryl‐1H‐pyrazole‐4‐carbaldehyde derivatives was synthesized using a green microwave‐assisted method.
- Structural confirmation was achieved through IR, ^1^H/^13^C NMR, and HRMS analyses.
- Anti‐inflammatory assays showed moderate to strong inhibition, with compounds 4c, 4d, and 4e being the most active against COX‐2.
- The compounds 4f and 4j exhibited sensitivity to TB at the MIC of 6.25 µg/mL, which was comparable to that of the standard drug pyrazinamide.
- Molecular docking with COX‐2 (PDB: 5KIR) supported the observed anti‐inflammatory results.

## Graphical abstract

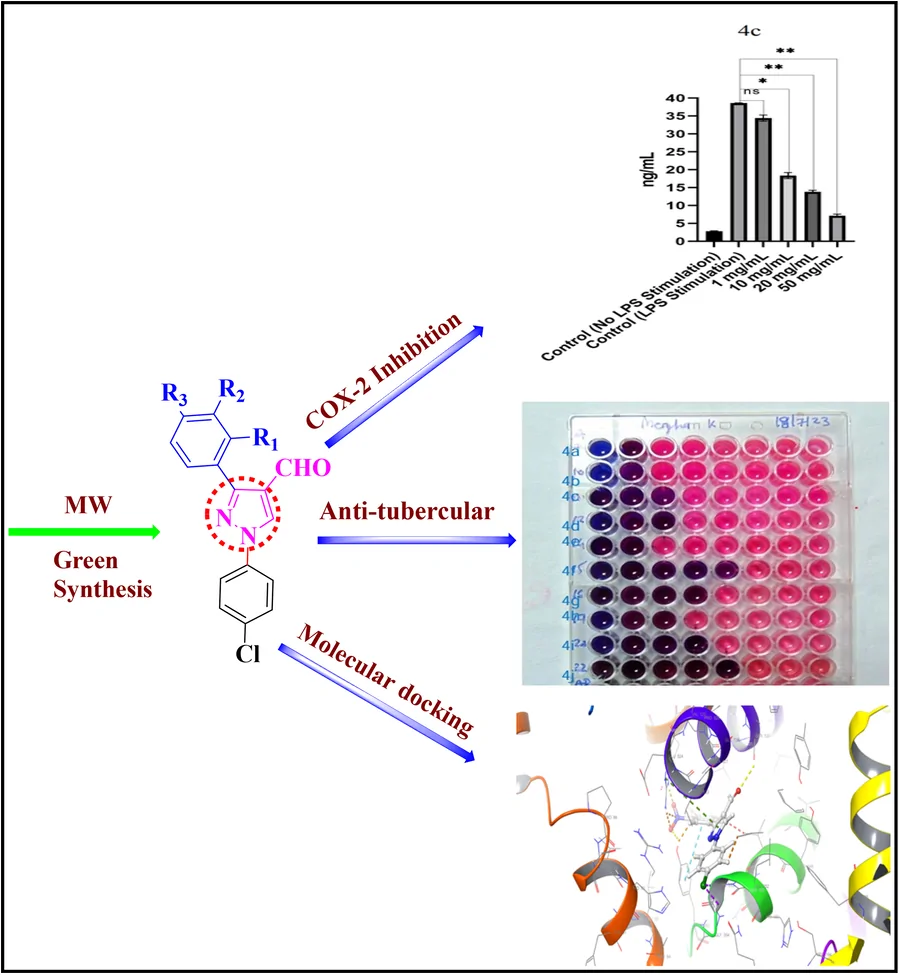

## 1 Introduction

Inflammation plays a crucial role in the development of various diseases, such as diabetes, heart disease, and some types of cancer. It represents the body's defense mechanism against microbial infections or tissue damage. Acute inflammation is characterized by pain, erythema, and edema, while chronic inflammation may contribute to conditions such as arthritis, asthma, and psoriasis. Cyclooxygenases are enzymes that facilitate the biosynthesis of prostaglandins, mediators involved in inflammation and other physiological processes. Nonsteroidal anti‐inflammatory drugs (NSAIDs) block the enzymes Cyclooxygenase‐1 (COX‐1) and Cyclooxygenase‐2 (COX‐2), thereby reducing prostaglandin synthesis and mitigating inflammation. However, long‐term NSAID use has been linked to negative health effects, including gastrointestinal mucosal damage, bleeding, intolerance, and kidney toxicity resulting from the non‐selective suppression of both COX isoforms. Therefore, there is a clear need for drugs that selectively inhibit COX‐2. While the Coxibs class of drugs provides selective COX‐2 inhibition and is generally safer for the gastrointestinal tract, they may pose significant risks to the cardiovascular system [1]. Consequently, evaluating the COX‐2 inhibitory profile of potential drugs is of paramount importance in pharmacological development.

Tuberculosis (TB) is a significant worldwide health issue and one of the most important chronic communicable diseases, primarily caused by Mycobacterium TB [2]. Despite major advancements in medical science, it continues to be a leading cause of death globally. The 2021 Global TB Report by the WHO states that more than 5.8 million new TB cases were recorded worldwide, resulting in 1.3 million deaths among HIV‐negative individuals and 214,000 deaths in those co‐infected with HIV [3]. The development of effective TB treatments has been hindered by the emergence of drug‐resistant strains, posing a substantial challenge to global efforts to combat the disease. Although many drug candidates show early potential, the increasing drug resistance in *M. tuberculosis* complicates treatment strategies, highlighting the urgent need for new therapeutic solutions. This manuscript aims to explore the challenges in TB drug development and the ongoing efforts to overcome drug resistance in the management of TB [4].

Previous literature showed that “Pyrazole core” had a wide spectrum of biological activities such as anti‐inflammatory [5, 6, 7, 8, 9, 10], anti‐tubercular [11], anti‐diabetics [12], ant‐parasitic agent [13], anti‐cancer [14, 15, 16, 17], aldose‐reductase inhibitor [18] anti‐microbial [19, 20, 21, 22, 23], apoptosis inducers [24], acetylcholinesterase inhibitors [25], insecticidal [26], etc. The pyrazole scaffold has emerged as a promising structural motif in anti‐tubercular medicinal chemistry, with recent reports of 1,3‐disubstituted pyrazoles achieving minimum inhibitor concentration (MIC) values in the low µg/mL range against *M. tuberculosis* H37Rv and other strains [27, 28, 29]. Concurrently, inflammatory responses in TB involve COX‐2–mediated prostaglandin E_2_ (PGE_2_) production in macrophages and dendritic cells, which influences the host immune response to infection [30, 31]. Therefore, the present work aimed to explore pyrazole derivatives both for direct anti‐Mtb activity and for COX‐2 inhibitory potential, hypothesizing that such dual‐acting agents might modulate not only pathogen replication but also host inflammatory pathways relevant to TB pathogenesis.

Based on the information mentioned above, we aimed to synthesize novel Pyrazole‐4‐carbaldehyde derivatives by the Vilsmeier‐Haack reaction. For this purpose, we tried the traditional method as well as the microwave irradiation method (MW). Microwave irradiation delivered a higher yield in less time than the traditional method. Chemists may now create desired organic compounds and catalytic materials or nanomaterials selectively, with nearly quantitative quantities, and with more precision than they could with traditional heating, thanks to significant advancements in MW‐assisted chemistry. Researchers can now proceed to the next stage of enhanced nanomaterial design and development by regulating the particular MW parameters (temperature, pressure, and temperature ramping) and solvent selection. Additionally, the synthesized 1‐(4‐Chorophenyl)‐3Aryl‐1‐*H*‐pyrazole‐4‐carbaldehyde derivatives were investigated for their potential biological application in the determination of anti‐inflammatory and anti‐tubercular potential, along with molecular docking parameters.

## 2 Results and Discussion

### 2.1 Chemistry

**Scheme 1:**
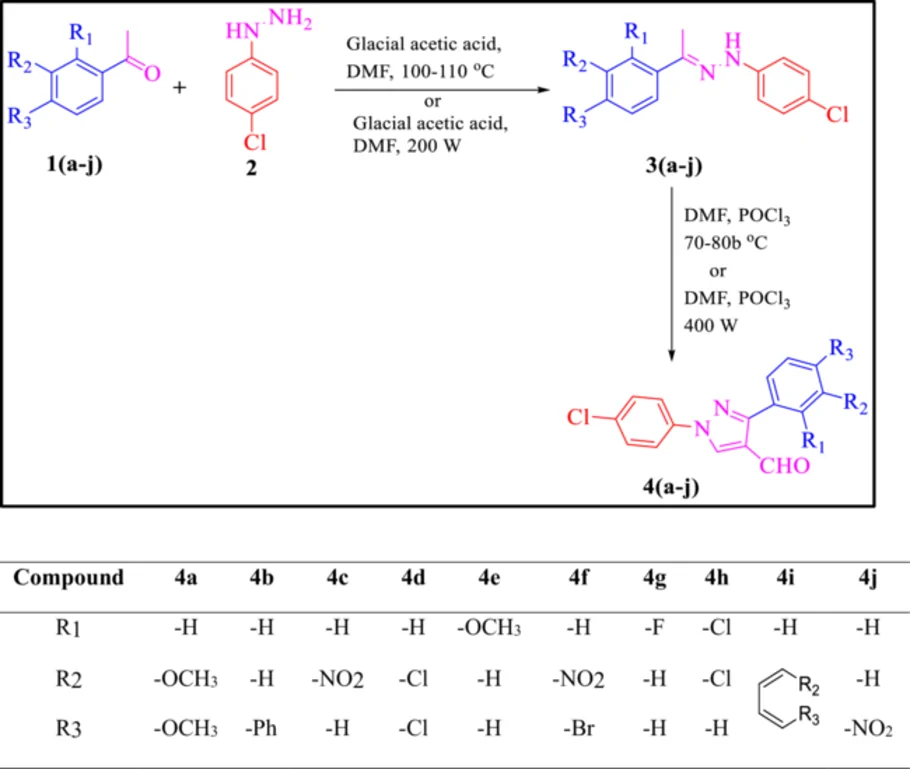
Syntheses of Pyrazole‐4‐carbaldehydes **4a–j**.

**Scheme 2:**
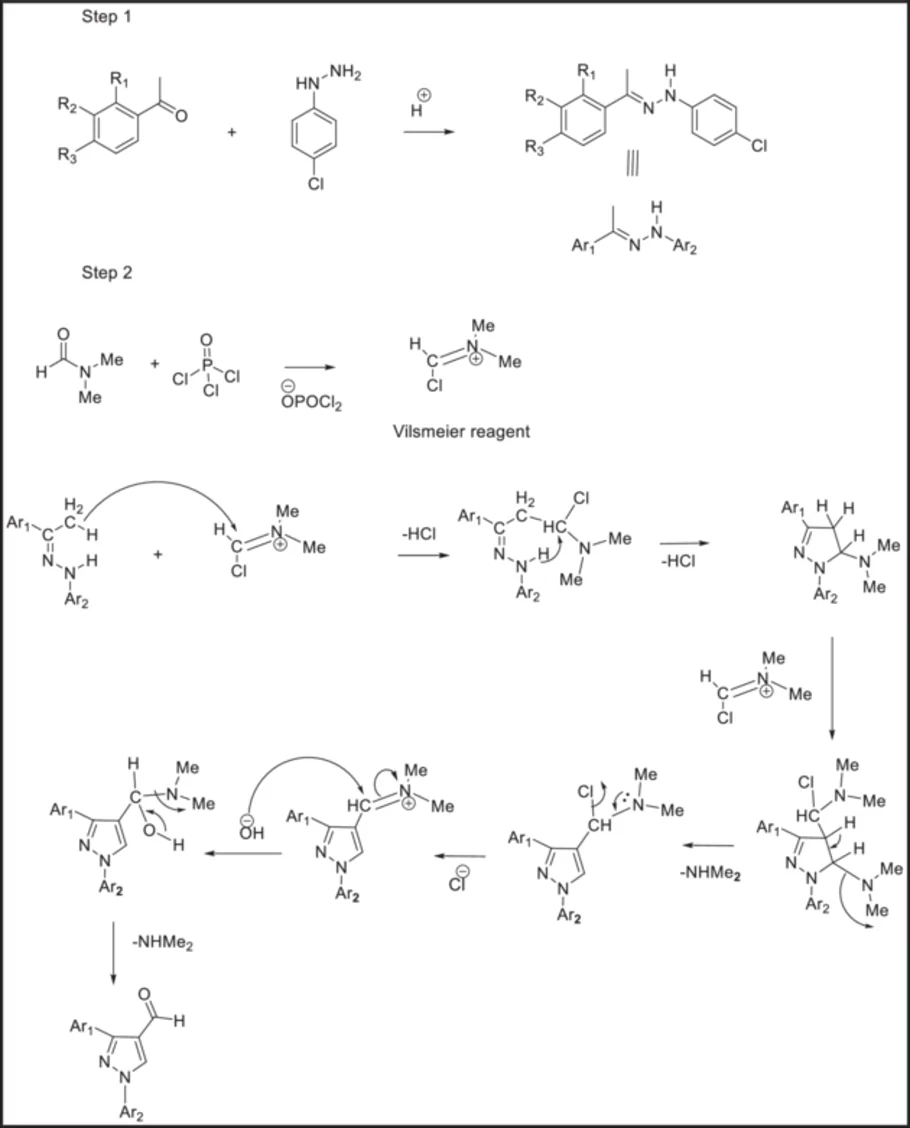
Plausible mechanism for the synthesis of Pyrazole‐4‐carbaldehydes **4a–j**.

In the continuation of our efforts towards green chemistry, we chose an environmentally benign, highly efficient microwave‐assisted method. To study the scope of the present method, we prepared 4a‐j using traditional heating as well as the microwave irradiation process. Microwave irradiation accelerates each step by efficiently heating polar molecules, leading to shorter reaction times, higher yields, and cleaner conversions compared to conventional methods. Microwave irradiation provides rapid and uniform heating, allowing polar reactants and intermediates to absorb energy efficiently, which accelerates the condensation and cyclization steps involved in pyrazole ring formation. Mechanistically, the microwave energy enhances the collision frequency and orientation of reactive species, promoting faster nucleophilic attack and cyclization, leading to higher yields in shorter reaction times. The synthesis of a series of 1‐(4‐Chlorophenyl)‐3‐Aryl‐1*H*‐pyrazole‐4‐carbaldehydes 4a‐j was carried out by a recently reported microwave‐assisted method [32]. This method involves two steps, as shown in Scheme 1. The reaction proceeds via a microwave‐assisted Vilsmeier–Haack formylation (Scheme 2). Initially, a substituted acetophenone 1a‐j condenses with 4‐chlorobenzyl hydrazine to form a hydrazone 3a‐j intermediate through nucleophilic attack and dehydration. Meanwhile, DMF and POCl_3_ react to generate the Vilsmeier reagent ${\left[\mathrm{ClCH}=N{\left(\mathrm{Me}\right)}_{2}\right]}^{+},$ a key electrophile. This reagent attacks the electron‐rich carbon of the hydrazone or aromatic substrate, forming an iminium intermediate that, upon hydrolysis, yields the formylated product 4a‐j.

The comparative data clearly demonstrate the superior efficiency of the MW method over the conventional thermal approach (Table 1). In the traditional method, the reactions required approximately 5.5–6.5 h to complete, giving moderate yields ranging from 58% to 67% for compounds 4a–j. In contrast, under microwave irradiation, the same transformations were accomplished within only 7–9 min, indicating a dramatic reduction in reaction time. Moreover, the product yields have improved significantly, reaching 70‐90%, with most compounds showing an increase of 10%–25% compared to the conventional method. This improvement can be attributed to the uniform and rapid heating provided by microwave energy, which enhances molecular collisions and reaction kinetics, minimizes side reactions, and ensures cleaner product formation. Overall, the MW technique proves to be a highly efficient, time‐saving, and yield‐enhancing alternative to the traditional method for the synthesis of compounds 4a–j.

**Table 1:** Physical data of the synthesized Pyrazole‐4‐carbaldehydes 4a–j.

Compounds	Traditional method		MWI method	
	Time (h)	Yield (%)	Time (min)	Yield (%)
**4a**	6	65	7	75
**4b**	6	66	8	80
**4c**	6.5	60	8	70
**4d**	6	64	8	87
**4e**	6	62	7	88
**4f**	6	60	9	75
**4g**	5.5	67	7	90
**4h**	6	59	8	78
**4i**	6.5	58	9	81
**4j**	6	61	8	80

**Figure 1:**
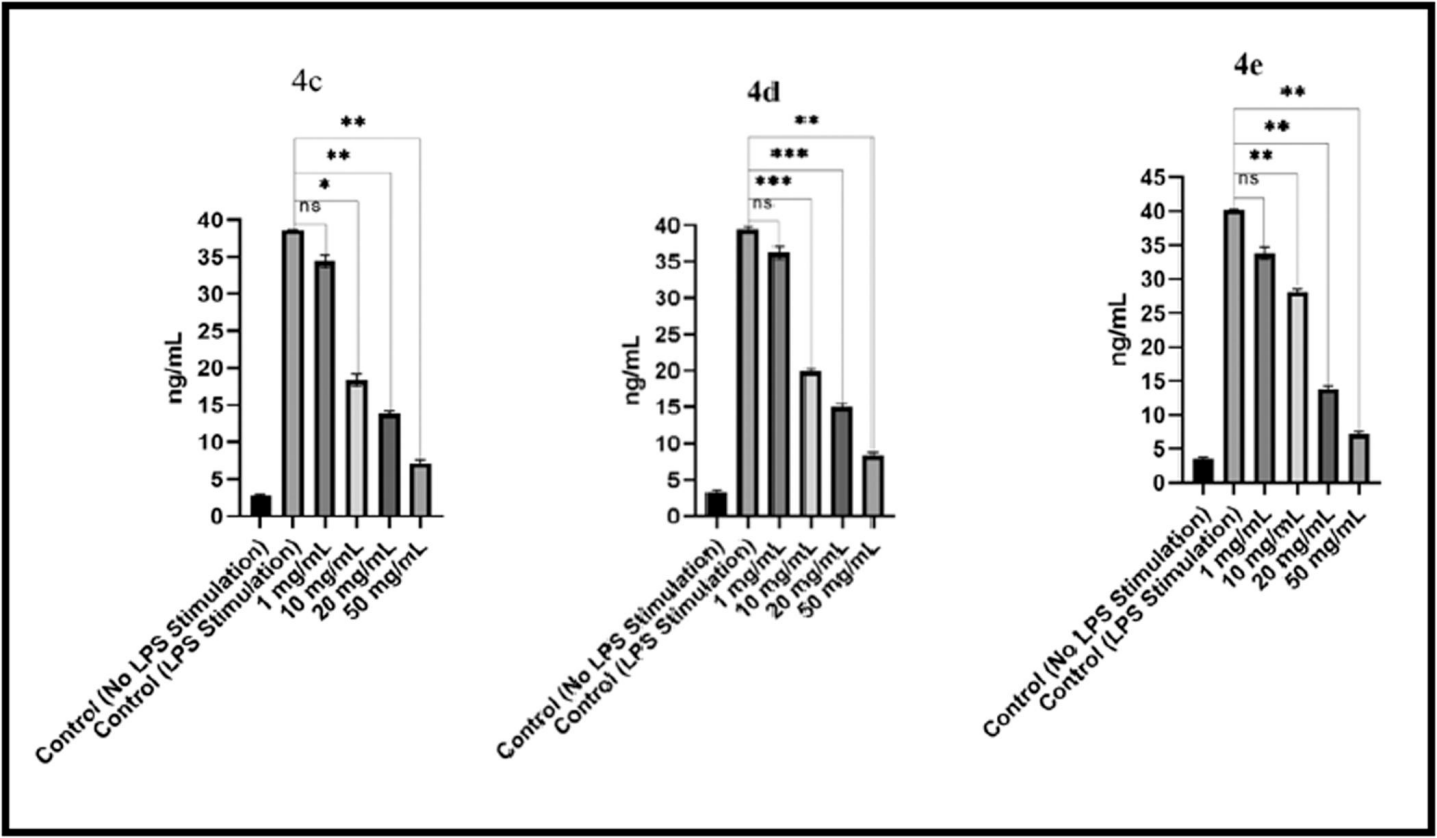
Inhibition of COX‐2 concentration by 4c, 4d, and 4e at various concentrations. ns = not significant; *p < 0.05, **p < 0.01, ***p < 0.001.

The formation of the product was confirmed by spectral analysis (supplementary files). The IR spectra around 1680 cm^−1^ of the C═O stretching frequency and C─H stretching around 2840 cm^−1^ indicate the presence of an aldehyde group (─CHO). Furthermore, no absorption of N─H stretching around 3350 cm^−1^ and N─H bending around 1350 cm^−1^ confirms the expected structure of the product. The ^1^H‐NMR of compounds showed a characteristic singlet of aldehydic proton around δ 10 ppm, also there was singlet around 8.5 ppm due to pyrazole‐*H*, confirming pyrazole ring formation. In ^13^C‐NMR spectra, the signal around δ 185 ppm indicates the presence of the formyl group. HRMS (ESI) also shows (M + 1) intense peak, which exactly matches the theoretically calculated mass from the molecular formula. All spectral analysis results are consistent with the expected structure of the product (Supporting Information S1: Figure 1–39).

### 2.2 Biological Activity

#### 2.2.1 Anti‐Inflammatory Activity

**Figure 2:**
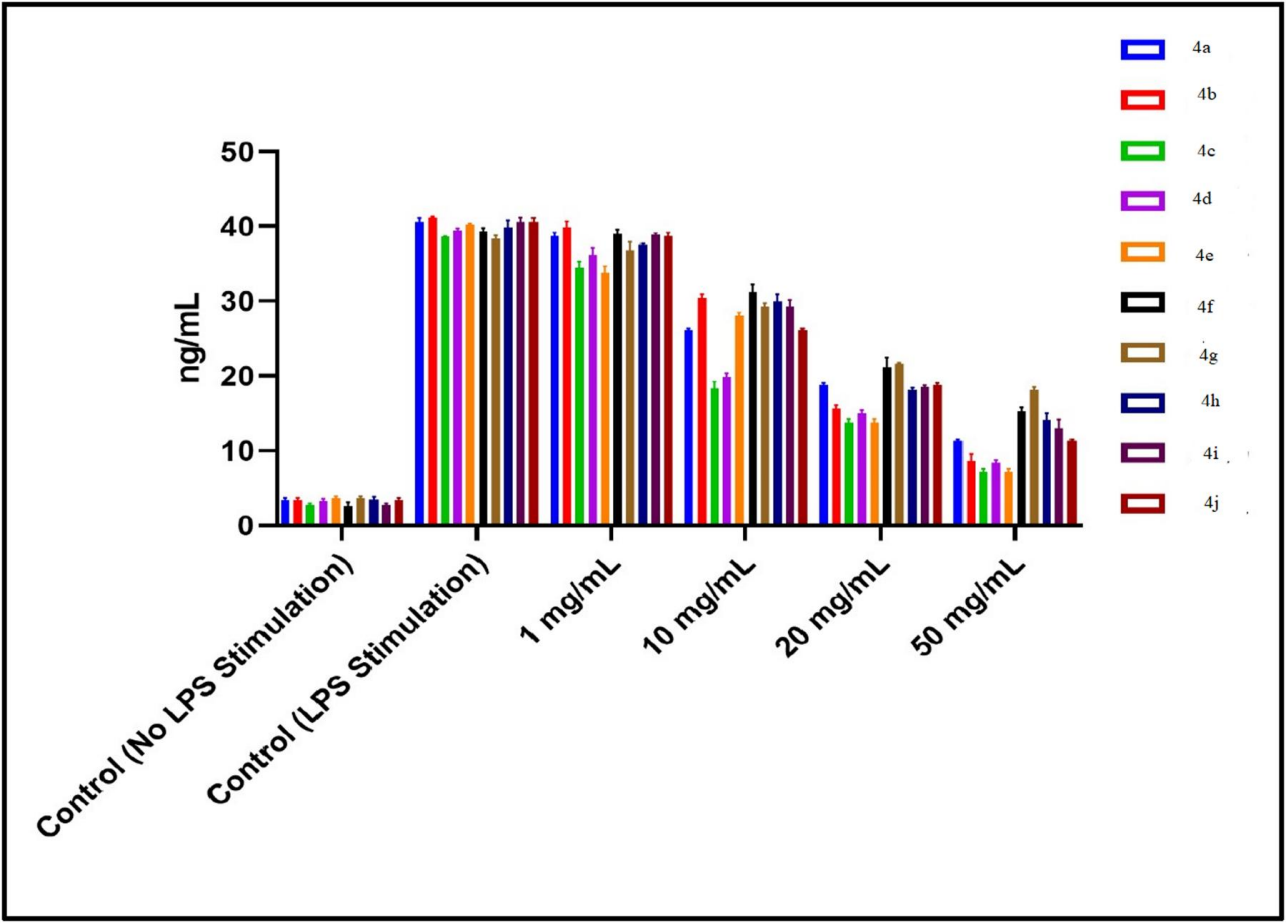
Inhibition of COX‐2 concentration by Pyrazole‐4‐carbaldehydes 4a–j.

In this study, the newly synthesized compounds **4a–j** were evaluated for their anti‐inflammatory activity [33, 34, 35, 36]. The COX‐2 enzyme plays a critical role in the production of pro‐inflammatory prostaglandins, making it an attractive target for anti‐inflammatory drug development. The THP‐1 cell line, a human monocytic cell line, was employed due to its ability to differentiate into macrophage‐like cells, which express COX‐2 upon stimulation. The cell supernatant was utilized as a source of COX‐2 protein for the ELISA assay, providing insights into the ability of the synthesized compounds to modulate LPS‐induced COX‐2 protein levels. The experiment included control conditions with and without LPS stimulation along with multiple concentrations of the compound under testing (1, 10, 20, and 50 mg/mL) as shown in Figure 2. The control condition without LPS stimulation consistently exhibited lower COX‐2 levels compared to the control condition with LPS stimulation, confirming the expected upregulation of COX‐2 protein expression following LPS treatment. IC_50_ values were determined as the concentration of each compound required to inhibit 50% of LPS‐induced COX‐2 protein levels. Percent inhibition for each replicate was calculated relative to LPS‐stimulated and unstimulated controls. IC_50_ for each replicate was estimated by linear interpolation between the two concentrations surrounding 50% inhibition. The mean IC_50_ and standard deviation (SD) for each compound were calculated from replicate IC_50_ values to reflect experimental variability. Compounds for which 50% inhibition was not reached within the tested range were noted as extrapolated. When tested at different concentrations, compound **4a‐j** exhibited a concentration‐dependent reduction in COX‐2 protein levels. At the lowest concentration (1 mg/mL), there was minimal reduction in inflammation, suggesting weak modulation of LPS‐induced COX‐2 expression. As the concentration increased to 10 mg/mL, the anti‐inflammatory effect became more noticeable, with a moderate reduction in the inflammatory markers. The 20 mg/mL concentration showed a more significant decrease in inflammatory values, approaching those of the non‐LPS‐stimulated control, indicating stronger suppression of LPS‐induced COX‐2 expression. The highest concentration (50 mg/mL) provided the most pronounced reduction in inflammation, with values consistently lower than those observed in the LPS‐stimulated control, suggesting potent COX‐2 inhibition. Overall, the data reveal a clear dose‐response relationship, with higher concentrations of the substance leading to more substantial inhibition of COX‐2 and a greater reduction in LPS‐induced COX‐2 protein levels. The strongest anti‐inflammatory effects were observed at 50 mg/mL, highlighting the potential of these compounds to suppress LPS‐induced COX‐2 expression. Among the tested derivatives, compounds **4c**, **4d**, and **4e** exhibited significant reductions in COX‐2 protein levels across all concentrations (Figure 1).

Structural variations were introduced at three positions of the aromatic ring (R_1_, R_2_, and R_3_) to investigate their influence on biological activity. The inhibitory potencies of the synthesized compounds were evaluated in vitro, and the resulting IC_50_ values are summarized in Table 2. The biological evaluation revealed distinct trends relating substituent type and position to potency. Among the series, compounds **4c** (IC_50_ = 7.78 mg/mL) and **4e** (IC_50_ = 7.89 mg/mL) exhibited the highest activity. These two derivatives highlight the favorable influence of electronic modulation at R_2_ and R_1_, respectively. In compound **4c**, the strongly electron‐withdrawing nitro group at R_2_ appears to enhance the interaction of the hydrazone moiety with the target site, likely by stabilizing the imine linkage and increasing the overall electrophilicity of the scaffold. Conversely, the improved activity of **4e** indicates that electron donation at R_1_, through the methoxy group, also contributes positively, possibly by reinforcing resonance stabilization or optimizing aromatic electron density for π–π stacking interactions. Substitution at the R_2_ position was found to be particularly influential. Electron‐withdrawing groups such as nitro (**4c**) and chloro (**4d**) generally enhanced activity compared to unsubstituted analogues. However, the beneficial effect of R_2_ substitution was strongly dependent on the steric environment at R_3_. When the nitro group at R_2_ was paired with a bulky substituent at R_3_, as in compound **4f** (R_3_ = Br), a dramatic reduction in activity was observed (IC_50_ = 31.62 mg/mL). This outcome suggests that while electronic withdrawal at R_2_ is favorable, excessive steric congestion near the hydrazone linkage disrupts optimal binding orientation. The R_3_ position tolerated moderate steric bulk. Compound **4b** (R_3_ = phenyl) demonstrated good activity (IC_50_ = 8.81 mg/mL), indicating that aromatic substitution may participate in additional stabilizing interactions such as π–π stacking within the active site. In contrast, compounds containing heavy halogens at R_3_ combined with strong EWGs at R_2_ (**4f**) showed significantly diminished potency, highlighting the importance of steric balance at this position. Halogenation effects were also positional. Compound **4d** (R_2_ = R_3_ = Cl) maintained reasonable activity (IC_50_ = 9.43 mg/mL), demonstrating that halogens in these positions enhance hydrophobic interactions without introducing excessive steric hindrance.

**Table 2:** IC_50_ values of COX‐2 enzyme inhibition by Pyrazole‐4‐carbaldehydes 4a–j.

Entry	IC_50_ value (mg/mL)
**4a**	13.30 ± 0.04
**4b**	8.81 ± 0.05
**4c**	7.78 ± 0.06
**4d**	9.43 ± 0.07
**4e**	7.89 ± 0.05
**4f**	31.62 ± 0.12
**4g**	>50
**4h**	16.69 ± 0.08
**4i**	30.76 ± 0.10
**4j**	13.30 ± 0.04

Overall, the structure‐activity relationship derived from this series indicates that optimal activity is achieved with small or moderately sized substituents at R_3_, strongly electron‐withdrawing groups at R_2_, and either electron‐neutral or mildly electron‐donating groups at R_1_. Substituent patterns that introduce significant steric hindrance or impose conflicting electronic effects across the aromatic ring result in notable attenuation of activity, as seen in compounds **4f, 4i,** and **4g**. These findings collectively provide a clear rationale for further structural optimization and highlight key regions of the scaffold that can be modified to enhance biological performance. However, additional studies are required to evaluate its safety and efficacy in clinical settings.

#### 2.2.2 Anti‐Tubercular Activity

Anti‐mycobacterial activity of synthesized compounds against *M. tuberculosis* was tested using the non‐toxic, heat‐stable Microplate Alamar Blue Assay (MABA), and MIC was defined as the lowest concentration that prevented the color change from blue to pink, which aligns well with methods such as BACTEC [37, 38, 39, 40]. In MABA, although bacterial growth inhibition is assessed via a redox indicator rather than turbidity, the assay follows CLSI‐aligned criteria, including standardized inoculum size, serial twofold dilutions of compounds, appropriate growth and sterility controls, and incubation under controlled conditions. Thus, MABA serves as a reliable surrogate for CLSI‐recommended broth microdilution methods, particularly for slow‐growing organisms such as *M. tuberculosis*. The results of the anti‐TB activity tests show that the compounds exhibit varying levels of efficacy at different concentrations. At the highest concentration (100 µg/mL), all compounds (**4a to 4j**) demonstrate sensitivity, indicating strong antibacterial activity at this concentration. However, as the concentration was decreased, differences in the activity profiles of the compounds became apparent. For example, compounds **4a, 4b,** and **4e** remain susceptible at 100 and 50 µg/mL but become resistant at concentrations below 50 µg/mL. Compounds **4c, 4d**, and **4h** exhibit a similar pattern, retaining activity at higher concentrations but showing resistance at concentrations below 25.0 µg/mL. Whereas compounds **4g** and **4i** showed activity up to 12.5 µg/mL, with MIC values of 12.50 µg/mL. In contrast, compounds **4f** and **4j** exhibited the highest activity among the synthesized compounds, remaining active even at 6.2 µg/mL, with MIC values of 6.25 µg/mL. This suggests that these compounds have a broader range of activity compared to others. The reference drugs exhibited higher anti‐mycobacterial activity than the synthesized compounds. Pyrazinamide showed an MIC of 3.125 µg/mL, while isoniazid and ethambutol exhibited MIC values of 1.60 µg/mL. Streptomycin and rifampicin showed the highest activity, with MIC values of 0.80 µg/mL. Although compounds **4f** and **4j** were the most active compounds in the synthesized series, their MIC values were higher than that of pyrazinamide. Nevertheless, their ability to inhibit *M. tuberculosis* at 6.25 µg/mL indicates that **4f** and **4j** are promising compounds for further investigation. Further studies are needed to assess their efficacy in vivo and to evaluate their safety and pharmacokinetic profiles for potential therapeutic use. Here, from the results, it was observed that electron‐donating groups or atoms at 2, 3, and/4 positions decreased anti‐TB activity. On the other hand, the electron‐withdrawing group or atom at position‐4 (**4j**) increases anti‐TB activity more than the same group or atom at position‐2 (**4c**). Among all the compounds tested **4f** (3‐NO_2_, 4‐Br) and **4j** (4‐NO_2_) have good anti‐tubercular activity as shown in Table 3.

**Table 3:** Anti‐tubercular activity of Pyrazole‐4‐carbaldehydes 4a‐j.

Sr. no.	Compounds	MIC (µg/mL)
1	**4a**	50
2	**4b**	50
3	**4c**	25
4	**4d**	25
5	**4e**	50
6	**4f**	6.25
7	**4g**	12.50
8	**4h**	25
9	**4i**	12.50
10	**4j**	6.25
11	Pyrazinamide	3.125
12	Isoniazid	1.60
13	Ethambutol	1.60
14	Streptomycin	0.80
15	Rifampicin	0.80

### 2.3 Molecular Docking

**Figure 3:**
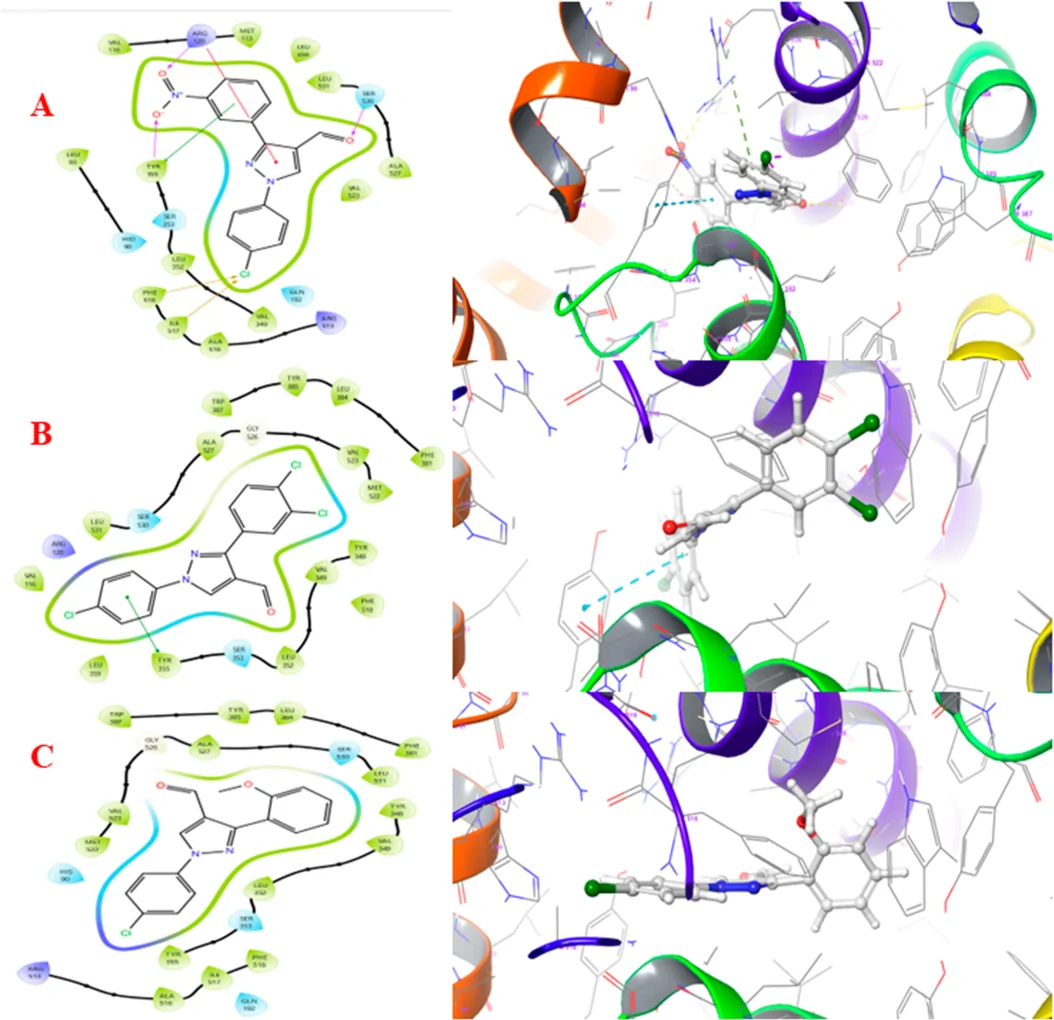
2D and 3D Binding pose and docking interaction of (A) **4c**, (B) **4d,** and (C) **4e** with human COX‐2.

Molecular docking studies were conducted to complement the biological screening results for anti‐inflammatory activity. The ligands were docked against the active site of human COX‐2 (PDB ID: 5KIR) [41, 42]. The docking analysis was performed using AutoDock Vina in UCSF Chimera 1.19 (Table 4). The docking protocol was validated through redocking studies, with the root‐mean‐square deviation (RMSD) calculated to be 2.050 Å. This value falls within the generally accepted threshold (≤ 2.0–2.5 Å) for reliable reproduction of the experimental binding mode, indicating that the docking procedure is suitably accurate. Although slightly above the ideal value of 2.0 Å, it remains well within the acceptable range, suggesting that the predicted ligand pose closely matches the experimental conformation with only minor deviations. This level of agreement supports the reliability of the docking protocol for subsequent binding‐mode analysis and comparative studies. Among the tested compounds, derivative **4c** exhibited the most favorable binding affinity with a docking score of –8.6 kcal/mol, indicating strong predicted affinity toward COX‐2. Detailed interaction revealed that **4c** interacts with COX‐2 via H‐bonds with TYR355, SER530, and ARG120, which are known to play essential roles in ligand anchoring near the entrance of the catalytic pocket. A Pi‐Pi Stacking interaction with TYR355, a halogen bond with PHE518, and an ILE517 further reinforce the ligand's orientation within the hydrophobic channel, while Pi cation interaction with ARG120 enhances the electrostatic complementarity. Additional polar contacts with SER353 and SER530 help to maintain the ligand in an optimal binding pose, consistent with a strong inhibitory potential. Derivative **4d** showed a binding energy of −7.4 kcal/mol, Pi‐Pi stacking with TYR355, polar interactions with SER530 and SER353, and a charged positive interaction with ARG120. Derivative **4e** exhibited a binding energy of −8.3 kcal/mol with charged and polar contact with ARG513, HIS90, SER530, SER353. (Figure 3).

**Table 4:** Binding energy of synthesized Pyrazole carbaldehydes 4a‐j against human COX‐2.

Sr. no.	Compounds	Binding affinity
1	**4a**	−7.3
2	**4b**	−8.4
3	**4c**	−8.6
4	**4d**	−7.4
5	**4e**	−8.3
6	**4f**	−7.6
7	**4g**	−8.6
8	**4h**	−9.0
9	**4i**	−8.5
10	**4j**	−8.1

The results also highlight that different derivatives engage in distinct interaction patterns, including amide‐Pi stacking and polar interactions with various residues, such as TYR355, ARG120, SER530, and GLN192. These interactions are crucial for stabilizing the ligand‐enzyme complex and enhancing its inhibitory potential. Synthesized compounds show promise as COX‐2 inhibitors and could potentially be developed into therapeutic agents for inflammation‐related disorders. Further experimental validation, including enzyme inhibition assays and in vivo studies, would be necessary to confirm their efficacy and safety profiles.

## 3 Conclusion

A new series of 1‐(4‐chlorophenyl)‐3‐aryl‐1*H*‐pyrazole‐4‐carbaldehyde derivatives was successfully synthesized using an efficient microwave‐assisted method, and their structures were confirmed through comprehensive spectroscopic analyses. Biological evaluation revealed that compounds **4c** and **4e** possessed notable reduction of LPS‐induced COX‐2 protein levels, while compounds **4f** and **4j** demonstrated promising anti‐tubercular activity, exhibiting potency comparable to the standard drug pyrazinamide. Molecular docking studies against the COX‐2 active site further corroborated the experimental findings, indicating favorable binding interactions consistent with their observed anti‐inflammatory potential. Overall, these results suggest that the synthesized pyrazole derivatives serve as promising lead molecules for the development of future anti‐inflammatory and anti‐tubercular therapeutic agents. These findings collectively provide a clear rationale for further structural optimization and highlight key regions of the scaffold that can be modified to enhance biological performance. However, further experimental validation, including enzyme inhibition assays and in vivo studies, would be necessary to confirm their efficacy and safety profiles.

## 4 Experimental

### 4.1 Chemistry

#### 4.1.1 General

The chemicals used in this study were sourced from Sigma‐Aldrich and were applied without any additional purification. Melting points for all newly synthesized compounds were determined using an open glass capillary, without any corrections. ^1^H and ^13^C‐NMR spectra were acquired on a Bruker Ultra Shield (400 MHz) NMR spectrometer. Infrared spectra of the compounds were recorded using a Bruker FTIR spectrometer, with a range of 4000–500 cm^−1^. The molecular weights of the synthesized compounds were confirmed by the molecular ion peaks (m/z) in the HRMS (ESI) spectrum, obtained using a Bruker Maxis Impact (Q‐TOF B9041) system.

#### 4.1.2 General Procedure for the Synthesis of Phenyl Hydrazones 3a–j

a. **Traditional method:**
  A mixture of one of the acetophenones 1a‐j (0.01 mol) and 4‐chlorophenylhydrazine (2) (0.01 mol) was dissolved in DMF (5 mL) with a few drops of glacial acetic acid and refluxed for 2–3 h. upon completion of the reaction, the mixture was poured into crushed ice, leading to the formation of a solid product. This solid was subsequently filtered to separate it from the solution. To ensure purity, the solid was washed with water to remove any soluble impurities, then dried to eliminate residual moisture. Finally, recrystallization from ethanol was performed, further purifying the product and yielding the desired compound 3a‐j. This method of purification was effective in obtaining a high‐quality product, which could be further analyzed for its properties and potential applications.
b. **Microwave irradiation method:**
  A mixture of acetophenone 1a‐j (0.01 mol) and 4‐chloro phenylhydrazine (2) (0.01 mol) in 0.5 mL DMF with 1–2 drops of CH_3_COOH was then exposed to microwave at 200 W for 3–4 min in 10‐s intervals, with reaction progress monitored by TLC. Upon completion of the reaction, the mixture was poured into crushed ice, leading to the formation of a solid product. This solid was subsequently filtered to separate it from the solution. To ensure purity, the solid was washed with water to remove any soluble impurities and then dried to eliminate residual moisture. Finally, recrystallization from ethanol was performed, further purifying the product and yielding the desired compound 3a‐j. This method of purification was effective in obtaining a high‐quality product, which could be further analyzed for its properties and potential applications.

#### 4.1.3 General Procedure for the Synthesis of 1‐(4‐chlorophenyl)‐3‐Aryl‐1*H*‐pyrazole‐4‐carbaldehydes (4a‐j)

a. **Traditional method:**
  To ice‐cold DMF (15 mL), phosphorus oxychloride (3 mL) was added dropwise with constant stirring, maintaining the temperature at 0°C to form the Vilsmeier‐Haack reagent. The hydrazone derivative **3a‐j** was then introduced into the mixture at 0°C, and the reaction was stirred at room temperature for 5 min. before being refluxed for 5–7 h. TLC monitored the reaction progress. upon completion of the reaction, the mixture was poured into crushed ice, leading to the formation of a solid product. This solid was subsequently filtered to separate it from the solution. To ensure purity, the solid was washed with water to remove any soluble impurities, then dried to eliminate residual moisture. Finally, recrystallization from ethanol was performed, further purifying the product and yielding the desired compound **4a‐j** [43, 44, 45, 46, 47, 48].
b. **Microwave irradiation method:**
  To ice‐cold DMF (15 mL), phosphorus oxychloride (3 mL) was added dropwise with constant stirring, maintaining the temperature at 0°C to generate the Vilsmeier‐Haack reagent. The hydrazone derivative 3a‐j was then added to the mixture at 0°C, followed by stirring at room temperature for 5 min. The reaction mixture was then subjected to microwave irradiation at 400 W for 7–9 min in 30‐s intervals, with the reaction progress monitored by TLC. Upon completion of the reaction, the mixture was poured into crushed ice, leading to the formation of a solid product. This solid was subsequently filtered to separate it from the solution. To ensure purity, the solid was washed with water to remove any soluble impurities and then dried to eliminate residual moisture. Finally, recrystallization from ethanol was performed, further purifying the product and yielding the desired compound 4a‐j.

##### 4.1.3.1 1‐(4‐chlorophenyl)‐3‐(3,4‐dimethoxyphenyl)‐1*H*‐pyrazole‐4‐carbaldehyde (4a)

White solid, Mp. 144°C, IR (KBr) cm^−1^: 3123 (Ar‐CH), 2833 (C─H in CHO), 1686 (C═O), 1500 (C═N), 790 (C─Cl). ^1^H‐NMR (400 MHz, CDCl_3_, δ ppm): 10.06 (S, 1H, ─CHO), 8.50 (S, 1H, pyrazole‐CH), 7.77–6.98 (m, 7H, Ar‐H), 3.98 (s, 3H, Ar‐OCH_3_), 3.96 (s, 3H, Ar‐OCH_3_). ^13^C─NMR (100 MHz, CDCl_3_, δ ppm): 185.0, 154.6, 150.1, 149.1, 137.4, 133.5, 131.4, 129.7, 123.7, 122.6, 121.8, 120.8, 111.6, 111.1, 112.0, 56.0, 55.9. HRMS (ESI, m/z): calcd. for C_18_H_15_N_2_O_3_Cl [M + H] + 343.0771; found 343.0848 [43].

##### 4.1.3.2 3‐([1,1′‐Biphenyl]‐4‐Yl)‐1‐(4‐Chlorophenyl)‐1*H*‐pyrazole‐4‐carbaldehyde (4B)

Brownish solid, Mp. 202°C, IR (KBr) cm^−1^: 3120(Ar‐CH), 2871(C─H in CHO), 1666 (C═O), 1559 (C═N), 824 (C─Cl). ^1^H‐NMR (400 MHz, CDCl_3_, δ ppm): 10.11 (S, 1H, ─CHO), 8.54 (S, 1H, pyrazole‐CH), 7.92‐7.26 (m, 13H, Ar‐H). ^13^C─NMR (100 MHz, CDCl_3_): 185.0, 154.5, 142.2, 140.3, 137.5, 133.6, 131.1, 130.0, 129.8, 129.3, 128.9, 127.7, 127.5, 127.1, 122.8, 120.8. HRMS (ESI, m/z): calcd. for C_22_H_15_N_2_OCl [M + H] + 359.0872; found 359.0951 [44].

##### 4.1.3.3 1‐(4‐chlorophenyl)‐3‐(3‐nitrophenyl)‐1*H*‐pyrazole‐4‐carbaldehyde (4c)

Brownish solid, Mp. 164°C, IR (KBr) cm^−1^: 3134(Ar‐CH), 2874 (C─H in CHO), 1682 (C═O), 1582 (C═N), 802 (C─Cl). ^1^H‐NMR (400 MHz, CDCl_3_, δ ppm): 10.08 (S, 1H, ─CHO), 8.845 (S, 1H, pyrazole‐CH), 8.841‐7.265 (m, 8H, Ar‐H). ^13^C─NMR (100 MHz, CDCl_3_): 183.4, 151.4, 148.4, 137.1, 134.7, 134.1, 133.3, 132.9, 129.9, 129.6, 124.0, 123.7, 122.8, 120.8. HRMS (ESI, m/z): calcd. for C_16_H_10_N_3_O_3_Cl [M + H] + 328.0410; found 328.0494.

##### 4.1.3.4 1‐(4‐chlorophenyl)‐3‐(3,4‐dichlorophenyl)‐1*H*‐pyrazole‐4‐carbaldehyde (4d)

Brownish solid, Mp. 190°C, IR (KBr) cm^−1^: 3114 (Ar‐CH), 2772 (C─H in CHO), 1679 (C═O), 1596 (C═N), 822 (C─Cl). ^1^H‐NMR (400 MHz, CDCl_3_, δ ppm): 10.04 (S, 1H, ─CHO), 8.52 (S, 1H, pyrazole‐CH), 8.04‐7.26 (m, 7H, Ar‐H). ^13^C─NMR (100 MHz, CDCl_3_): 183.8, 151.8, 137.2, 133.9, 133.7, 132.9, 132.5, 131.0, 130.7, 130.6, 130.5, 130.3, 129.9, 128.0, 127.3, 122.7, 120.8. HRMS (ESI, m/z): calcd. for C_16_H_9_N_2_OCl_3_ [M + H] + 350.9780 found 350.9858 [45].

##### 4.1.3.5 1‐(4‐chlorophenyl)‐3‐(2‐methoxyphenyl)‐1*H*‐pyrazole‐4‐carbaldehyde (4e)

White solid, Mp. 154°C, IR (KBr) cm^−1^: 3114 (Ar‐CH), 2826 (C─H in CHO), 1676 (C═O), 1581 (C═N), 785 (C─Cl). ^1^H‐NMR (400 MHz, CDCl_3_, δ ppm): 9.78 (S, 1H, ─CHO), 8.48 (S, 1H, pyrazole‐CH), 7.74–7.03 (m, 8H, Ar‐H), 3.08 (s, 3H, ─OCH_3_). ^13^C─NMR (100 MHz, CDCl_3_): 186.5, 156.8, 152.3, 137.7, 133.3, 131.2, 131.0, 129.7, 128.8, 123.6, 121.1, 120.7, 120.1, 111.1, 55.5. HRMS (ESI, m/z): calcd. for C_17_H_13_N_2_O_2_Cl [M + H] + 313.0665; found 313.0745.

##### 4.1.3.6 3‐(4‐bromo‐3‐nitrophenyl)‐1‐(4‐chlorophenyl)‐1*H*‐pyrazole‐4‐carbaldehyde (4f)

Yellowish solid, Mp. 172°C, IR (KBr) cm^−1^: 3117 (Ar‐CH), 2775 (C─H in CHO), 1675 (C═O), 1588 (C═N), 781 (C─Cl). ^1^H‐NMR (400 MHz, CDCl_3_, δ ppm): 10.06 (S, 1H, ─CHO), 8.58 (S, 1H, pyrazole‐CH), 8.57‐7.26 (m, 7H, Ar‐H). ^13^C─NMR (100 MHz, CDCl_3_): 183.0, 150.1, 149.9, 137.0, 135.1, 134.3, 134.2, 133.0, 131.9, 130.0, 125.6, 122.9, 120.8, 115.2.

##### 4.1.3.7 1‐(4‐chlorophenyl)‐3‐(2‐fluorophenyl)‐1*H*‐pyrazole‐4‐carbaldehyde (4g)

White solid, Mp. 166°C, IR (KBr) cm^−1^: 3124 (Ar‐CH), 2923 (C─H in CHO), 1679 (C═O), 1593 (C═N), 781 (C─Cl). ^1^H‐NMR (400 MHz, CDCl_3_, δ ppm): 9.80 (S, 1H, ─CHO), 8.51 (S, 1H, pyrazole‐CH), 7.73‐7.26 (m, 8H, Ar‐H). ^13^C─NMR (100 MHz, CDCl_3_): 184.5, 164.1, 161.6, 153.4, 153.3, 137.3, 133.8, 133.2, 133.1, 131.6, 130.4, 130.3, 129.8, 124.7, 124.6, 122.7, 120.8, 116.5, 116.2, 115.9, 115.7. HRMS (ESI, m/z): calcd. for C_16_H_10_N_2_OClF [M + H] + 301.0465; found 301.0544.

##### 4.1.3.8 1‐(4‐chlorophenyl)‐3‐(2,3‐dichlorophenyl)‐1*H*‐pyrazole‐4‐carbaldehyde (4h)

Brownish solid, Mp. 218°C, IR (KBr) cm^−1^: 3114 (Ar‐CH), 2925 (C─H in CHO), 1680 (C═O), 1597 (C═N), 820 (C─Cl). ^1^H‐NMR (400 MHz, CDCl_3_, δ ppm): 9.80 (S, 1H, ─CHO), 8.51 (S, 1H, pyrazole‐CH), 7.73‐7.26 (m, 7H, Ar‐H). ^13^C─NMR (100 MHz, CDCl_3_): 184.5, 151.4, 137.3, 136.1, 134.4, 133.8, 132.7, 129.9, 129.89, 129.84, 128.9, 127.4, 123.4, 120.9. HRMS (ESI, m/z): calcd. for C_16_H_9_N_2_OCl_3_ [M + H] + 350.9780; found 350.9868.

##### 4.1.3.9 1‐(4‐chlorophenyl)‐3‐(naphthalen‐2‐yl)‐1*H*‐pyrazole‐4‐carbaldehyde (4i)

White solid, Mp. 128°C, IR (KBr) cm^−1^: 3114 (Ar‐CH), 2846 (C─H in CHO), 1681 (C═O), 1597 (C═N), 820 (C─Cl). ^1^H‐NMR (400 MHz, CDCl_3_, δ ppm): 9.68 (S, 1H, ─CHO), 8.63 (S, 1H, pyrazole‐CH), 8.07‐8.26 (m, 12H, Ar‐H). ^13^C─NMR (100 MHz, CDCl_3_): 185.1, 154.8, 137.5, 133.7, 133.6, 133.2, 131.1, 129.8, 128.7, 128.6, 128.5, 128.4, 127.7, 126.9, 126.6, 126.1, 122.9, 120.9. HRMS (ESI, m/z): calcd. for C_20_H_13_N_2_OCl [M + H] + 333.0716; found 333.0791 [46].

##### 4.1.3.10 1‐(4‐chlorophenyl)‐3‐(4‐nitrophenyl)‐1*H*‐pyrazole‐4‐carbaldehyde (4j)

Yellowish solid, Mp.166°C, IR (KBr) cm‐1: 3120 (Ar‐CH), 1689 (C═O), 2835 (C─H in CHO), 1594 (C═N), 851 (C─Cl). ^1^H‐NMR (400 MHz, CDCl_3_, δ ppm): 10.08 (S, 1H, ─CHO), 8.56 (S, 1H, pyrazole‐CH), 8.35‐7.26 (m, 8H, Ar‐H). ^13^C─NMR (100 MHz, CDCl_3_): 146.4, 137.4, 135.0, 133.8, 130.0, 129.7, 123.8, 120.9, 65.5. HRMS (ESI, m/z): calcd. for C_16_H_10_N_3_O_3_Cl [M + H] + 328.0410; found 328.0502 [47, 48].

### 4.2 Biological Evaluation

#### 4.2.1 Anti‐Inflammatory Activity

THP‐1 cells were cultured at 37°C, 5% CO_2_, and 95% humidity in a suitable culture medium. The cells were maintained in a logarithmic growth phase, and approximately half a million cells were treated with each of the compounds. The compounds were filter‐sterilized before addition to ensure sterility and prevent potential contamination. The THP‐1 cells were treated with the compounds at 1, 10, 20, and 50 mg/mL concentrations for 24 h. The compounds were added directly to the cell culture medium, and the cells were incubated under the specified culture conditions. After the 24‐h incubation period, the cell culture plates were centrifuged at an appropriate speed and duration to separate the cells from the culture medium. Approximately 100 microliters of the cell supernatant from each well were collected for subsequent COX‐2 ELISA analysis. The Human COX‐2 ELISA Kit from Invitrogen was employed to measure COX‐2 concentrations in the cell supernatant. Absorbance readings were obtained using a microplate reader at a wavelength of 450 nm [49, 50, 51]. The calculated concentrations and OD values were analyzed using GraphPad Prism 8.4.2 software. Welch's test, a statistical test suitable for comparing two groups with potentially unequal variances, was performed to assess the significance of COX‐2 inhibition by the tested drugs. IC_50_ values were determined as the concentration of each compound required to inhibit 50% of LPS‐induced COX‐2 protein levels. Percent inhibition for each replicate was calculated relative to LPS‐stimulated and unstimulated controls. IC_50_ for each replicate was estimated by linear interpolation between the two concentrations surrounding 50% inhibition. The mean IC_50_ and SD for each compound were calculated from replicate IC_50_ values to reflect experimental variability. Compounds for which 50% inhibition was not reached within the tested range were noted as extrapolated.

#### 4.2.2 Anti‐Tubercular Activity

Anti‐mycobacterial activity of the synthesized compounds was evaluated using the MABA. To prevent evaporation of the medium from the test wells during incubation, 200 μL of sterile deionized water was added to all outer perimeter wells of a sterile 96‐well plate. The remaining wells were filled with 100 μL of Middlebrook 7H9 broth, and serial dilutions of the compounds were directly applied to the plate. The concentrations of the compounds ranged from 100 to 0.2 μg/mL. The plate was then covered, sealed with parafilm, and incubated at 37°C for 5 days. Following incubation, 25 μL of a freshly prepared 1:1 mixture of Alamar Blue reagent and 10% tween 80 was added to each well. The plate was then incubated for an additional 24 h before the results were assessed [38, 52, 53, 54, 55]. All tests were conducted in triplicate to ensure consistency and reliability of the results.

### 4.3 Molecular Docking Analysis

The docking study was conducted to evaluate the structure‐activity relationship. The ligand structure was drawn using ChemSketch into MOL format and further prepared for docking analysis. Molecular docking studies were conducted to complement the biological screening results for anti‐inflammatory activity. The ligands were docked against active site of human COX‐2 (PDB ID: 5KIR). The docking analysis was performed using AutoDock Vina in UCSF Chimera 1.19. Docking results were analyzed using the educational version of Maestro 14.5 [56, 57, 58].

## Author Contributions

**Megha Kokane:** conceptualization; writing – original draft. **Dnyandev Bhosale:** methodology; writing – review and editing. **Dattatraya Raut:** data curation. **Pushpa Hadimani:** investigation. **Ashwini Narale:** validation. **Prafulla Choudhari:** writing – review and editing; formal analysis. **Anjana Lawand:** project administration; supervision; visualization; writing – review and editing.

## Conflicts of Interest

The authors declare no conflicts of interest.

## Supporting information


Supporting File

## Data Availability

The data that support the findings of this study are available in the supplementary material of this article.
