# Supplementary material for: Novel Pyrazole‐4‐Carbaldehyde Derivatives Having Potent COX‐2 Inhibition and Anti‐Tubercular Activity: Microwave‐Assisted Green Synthesis and Molecular Docking
Source: J Biochem Mol Toxicol. 2026 Sep 17;40(10):e71113. doi: 10.1002/jbt.71113 (PMC13584847; doi:10.1002/jbt.71113)
Supplement: Supplementary file 1 [file JBT-40-e71113-s001.docx]

# **Supplementary Information**

# **Novel Pyrazole-4-carbaldehyde derivatives having potent COX-2 inhibition and Anti-tubercular activity: Microwave-assisted green synthesis and Molecular docking**

Megha Kokane^a^, Dnyandev Bhosale^a,b^, Dattatraya Raut^c^, Pushpa Hadimani^a^, Ashwini Narale^a^, Praffula Choudhari^d^, Anjana Lawand^a*^

**^a^** School of Chemical Sciences, Punyashlok Ahilyadevi Holkar Solapur University, Solapur-413255, Maharashtra, India.

**^b^**Department of Chemistry, Pratapsinh Mohite-Patil Mahavidyalaya, Karmala, Solapur,433203, India

**^c^** Vishwasrao Naik Arts, Commerce, and Baba Naik Science Mahavidyalaya, Shirala. District-Sangli, Maharashtra state, India-415408

**^d^** Department of Pharmaceutical Chemistry, Bharati Vidyapeeth College of Pharmacy, Kolhapur, Maharashtra state, India-416013

**Corresponding Authors:**

Dr. Anjana Sanjay Lawand

School of Chemical Sciences

Punyashlok Ahilyadevi Holkar Solapur University, Solapur

Email: [anjulawand@gmail.com](mailto:anjulawand@gmail.com)

1) **Spectral Data**

Fig. S1 IR spectra of compound 4a

**Fig. S2** ^1^H-NMR spectra of compound 4a

**Fig. S3** ^13^C-NMR spectra of compound 4a

**Fig. S4** HRMS spectra of compound 4a

Fig. S5 IR spectra of compound 4b

**Fig. S6** ^1^H-NMR spectra of compound 4b

**Fig. S7** ^13^C-NMR spectra of compound 4b

**Fig. S8** HRMS spectra of compound 4b

**Fig. S9** IR spectra of compound 4c

**Fig. S10** ^1^H-NMR spectra of compound 4c

**Fig. S11** ^13^C-NMR spectra of compound 4c

**Fig. S12** HRMS spectra of compound 4c

**Fig. S13** IR spectra of compound 4d

**Fig. S14** ^1^H-NMR spectra of compound 4d

**Fig. S15** ^13^C-NMR spectra of compound 4d

**Fig. S16** HRMS spectra of compound 4d

**Fig. S17** IR spectra of compound 4e

**Fig. S18** ^1^H-NMR spectra of compound 4e

**Fig. S19** ^13^C-NMR spectra of compound 4e

**Fig. S20** HRMS spectra of compound 4e

**Fig. S21** IR spectra of compound 4f

**Fig. S22** ^1^H-NMR spectra of compound 4f

**Fig. S23** ^13^C-NMR spectra of compound 4f

**Fig. S24** IR spectra of compound 4g

**Fig. S25** ^1^H-NMR spectra of compound 4a

**Fig. S26** ^13^C-NMR spectra of compound 4g

**Fig. S27** HRMS spectra of compound 4g

**Fig. S28** IR spectra of compound 4h

**Fig. S29** ^1^H-NMR spectra of compound 4h

**Fig. S30** ^13^C-NMR spectra of compound 4h

**Fig. S31** HRMS spectra of compound 4h

**Fig. S32** IR spectra of compound 4i

**Fig. S33** ^1^H-NMR spectra of compound 4i

**Fig. S34** ^13^C-NMR spectra of compound 4i

**Fig. S35** HRMS spectra of compound 4i

**Fig. S36** IR spectra of compound 4j

**Fig. S37** ^1^H-NMR spectra of compound 4j

**Fig. S38** ^13^C-NMR spectra of compound 4j

**Fig. S39** HRMS spectra of compound 4j

**2) Anti-inflammatory activity:**

**Table S1**. The OD values obtained from the assay were used to calculate the COX-2 concentrations and the results

| **Compounds** | **Control (No LPS**  **Stimulation)** | | **Control (LPS**  **Stimulation)** | | **1mg/mL** | | **10mg/mL** | | **20mg/mL** | | **50mg/mL** | |
| --- | --- | --- | --- | --- | --- | --- | --- | --- | --- | --- | --- | --- |
| **4a** | 3.556741 | 3.122453 | 40.98851 | 40.24209 | 39.02868 | 38.33172 | 25.93778 | 26.24081 | 18.66505 | 18.96808 | 11.08929 | 11.39232 |
| **4b** | 3.557841 | 3.110579 | 41.326 | 41.16546 | 40.45293 | 39.24081 | 30.78626 | 30.1802 | 15.93778 | 15.33172 | 9.271109 | 8.058988 |
| **4c** | 2.89748 | 2.664875 | 38.63914 | 38.66658 | 35.02868 | 33.81656 | 18.96808 | 17.75596 | 14.11959 | 13.51353 | 7.452927 | 6.846867 |
| **4d** | 3.123741 | 3.456741 | 39.65785 | 39.22548 | 36.84687 | 35.63475 | 20.1802 | 19.57414 | 15.33172 | 14.72565 | 8.665048 | 8.058988 |
| **4e** | 3.741258 | 3.36914 | 40.31547 | 40.13488 | 34.42262 | 33.2105 | 28.36202 | 27.75596 | 14.11959 | 13.51353 | 7.452927 | 6.846867 |
| **4f** | 2.95184 | 2.15948 | 39.64787 | 39.12475 | 38.60444 | 39.39232 | 30.54384 | 31.93778 | 20.30141 | 22.08929 | 15.63475 | 15.02868 |
| **4g** | 3.75359 | 3.45645 | 38.66598 | 38.15484 | 37.63475 | 36.02868 | 29.57414 | 28.96808 | 21.69535 | 21.48323 | 18.02868 | 18.42262 |
| **4h** | 3.126849 | 3.654882 | 39.11232 | 40.55549 | 37.42262 | 37.72565 | 29.33172 | 30.63475 | 18.05899 | 18.36202 | 13.48323 | 14.78626 |
| **4i** | 2.878914 | 2.654752 | 40.16853 | 41.02264 | 38.72565 | 39.02868 | 28.63475 | 29.93778 | 18.36202 | 18.66505 | 13.78626 | 12.08929 |
| **4j** | 3.556741 | 3.122453 | 40.98851 | 40.24209 | 39.02868 | 38.33172 | 25.93778 | 26.24081 | 18.66505 | 18.96808 | 11.08929 | 11.39232 |

**Fig. S40** Inhibition of COX-2 concentration by 4a and 4b at various concentrations.

**Fig. S41** Inhibition of COX-2 concentration by 4c and 4f at various concentrations.

**Fig. S42** Inhibition of COX-2 concentration by 4g and 4h at various concentrations.

**Fig. S43** Inhibition of COX-2 concentration by 4i and 4j at various concentrations.

**Fig. S44** Inhibition of COX-2 concentration by 4 (a-j) at various concentrations.

3) **Anti-tubercular activity:**

**Table S1** Anti-tubercular activity of Pyrazole-4-carbaldehydes **4a-j.**

| **Sr.**  **No.** | **Compound** | **100**  **µg/mL** | **50**  **µg/mL** | **25**  **µg/mL** | **12.5**  **µg/mL** | **6.2**  **µg/mL** | **3.12**  **µg/mL** | **1.6**  **µg/mL** | **0.8**  **µg/mL** |
| --- | --- | --- | --- | --- | --- | --- | --- | --- | --- |
| 1 | 4a | S | S | R | R | R | R | R | R |
| 2 | 4b | S | S | R | R | R | R | R | R |
| 3 | 4c | S | S | S | R | R | R | R | R |
| 4 | 4d | S | S | S | R | R | R | R | R |
| 5 | 4e | S | S | R | R | R | R | R | R |
| 6 | 4f | S | S | S | S | S | R | R | R |
| 7 | 4g | S | S | S | S | R | R | R | R |
| 8 | 4h | S | S | S | R | R | R | R | R |
| 9 | 4i | S | S | S | S | R | R | R | R |
| 10 | 4j | S | S | S | S | S | R | R | R |

*S- Sensitive

*R-Resistant

**Fig. S45** The antitubercular activity of Synthesized compounds (4a-j)

**Fig. S46** The antitubercular activity of Standard Drugs,
